# Combined association of walking pace and grip strength with incident type 2 diabetes

**DOI:** 10.1111/sms.14197

**Published:** 2022-06-03

**Authors:** Jirapitcha Boonpor, Solange Parra‐Soto, Fanny Petermann‐Rocha, Frederick K Ho, Carlos Celis‐Morales, Stuart R. Gray

**Affiliations:** ^1^ Institute of Cardiovascular and Medical Sciences University of Glasgow Glasgow UK; ^2^ Faculty of Public Health, Chalermphrakiat Sakon Nakhon Province Campus Kasetsart University Sakon Nakhon Thailand; ^3^ Institute of Health and Wellbeing University of Glasgow Glasgow UK; ^4^ Facultad de Medicina Universidad Diego Portales Santiago Chile; ^5^ Human Performance Lab, Education, Physical Activity and Health Research Unit University Católica del Maule Talca Chile

**Keywords:** gait, grip strength, relative excess risk due to interaction, type 2 diabetes mellitus, walking pace

## Abstract

The current study aims to investigate the combined association of walking pace and grip strength with incident type 2 diabetes (T2D). A total of 205 738 participants (mean age 56.6 ± 8.1 years, 115 139 [56.0%] women) without diagnosed or unknown diabetes at baseline from the UK Biobank study were included in this prospective study. Walking pace was self‐reported as slow, average, or brisk. Grip strength was measured using a dynamometer and classified as weak, average, and strong. The combined association of walking pace and grip strength with incident T2D was investigated using Cox‐proportional hazards models with a 2‐year landmark analysis. The additive interaction was conducted by estimating relative excess risk due to interaction (RERI). After the median follow‐up period of 5.4 years (interquartile range: 4.8–6.5), 5082 (2.5%) participants were diagnosed with T2D. Compared to brisk‐strong individuals (reference group), people who were slow‐weak had a higher risk of T2D (hazard ratio: 1.64 [95% CI, 1.42–1.89]) after adjusting for all covariates. There were dose–response gradients across both walking pace and grip strength variables. There was a modest amount of negative additive interaction (RERI; −0.06 [95% CI, −0.16; −0.01]. To conclude, slower pace and weaker grip strength were associated with a higher risk of developing T2D, independent of sociodemographics, lifestyle, and adiposity. Combining walking pace and grip strength might be a practical approach to screening people who are at increased risk of developing T2D.

## 1 INTRODUCTION

A wealth of evidence demonstrates that markers of physical capability such as walking pace and grip strength are associated with health outcomes. For example, walking pace and grip strength have both been associated with all‐cause mortality, cardiovascular disease (CVD), and cancer incidence and mortality.^1^, ^2^, ^3^, ^4^, ^5^, ^6^, ^7^, ^8^, ^9^, ^10^, ^11^, ^12^, ^13^ Emerging evidence has also shown that walking pace and grip strength are both associated with a higher risk of developing type 2 diabetes (T2D). For instance, a slower usual walking pace is associated with a higher risk of T2D incidence.^14^ Similarly, several studies have demonstrated that high muscular strength is associated with a lower risk of T2D incidence,^15^, ^16^, ^17^, ^18^, ^19^, ^20^ although some have reported the contrary.^4^, ^21^, ^22^ These inconsistent results have, however, recently been clarified in a meta‐analysis where it was demonstrated that each standard deviation (SD) higher muscular strength was associated with a 13% lower risk of T2D when controlling for adiposity, and a 24% lower risk when not controlling for adiposity.^23^

From a physiological point of view, walking pace and grip strength reflect different underlying physiological processes, grip strength is a simple contraction measuring strength whereas walking pace integrates strength with other processes such as balance and coordination, and so it is possible that the combination of both will result in a stronger association with health outcomes than individually. Indeed, previously we have demonstrated the additive effects of walking pace and grip strength for CVD risk prediction^24^ and sarcopenia.^13^ The combined associations of walking pace and grip strength with the risk of developing T2D has, to the best of our knowledge, not yet been investigated. The aim of the current study, therefore, is to investigate the combined association of walking pace and grip strength with incident T2D.

## 2 MATERIALS AND METHODS

### 2.1 Data source

The UK Biobank study recruited approximately 502 000 participants between 2006 and 2010 (5.5% response rate, men and women were aged 37–73 years) from the general population.^25^, ^26^ Participants attended 1 of 22 assessment centers across England, Wales, and Scotland.^26^, ^27^ Participants completed electronic consent, touch screen questionnaires, and physical measurements at the assessment centers, including grip strength and anthropometric measurements. Biological samples, including blood, urine, and saliva, were also collected as described elsewhere.^26^, ^27^ This present study included 205 738 participants. The exclusions were no data available from the primary care records (*n* = 273 991), participants who had diabetes (*n* = 12 967) or unknown diabetes (*n* = 1589) at baseline, and missing exposure or covariates (*n* = 8173).

### 2.2 Outcome

Incident T2D was derived from linkage to primary care data in the UK Biobank. Records were extracted for 45% of the UK Biobank cohort (228 467 participants). The end of coverage (extract date) was May 2017 for Scotland, September 2017 for Wales, and August 2017 for England. Detailed linkage procedures are available at http://biobank.ndph.ox.ac.uk/showcase/showcase/docs/primary_care_data.pdf. We defined incident T2D as primary care diagnosed with ICD‐10 (International classification of diseases, 10^th^ revision) code E11. READ codes used in the primary care data were converted into ICD‐10 codes using UK Biobank's look‐up table.

### 2.3 Exposures

Participants self‐reported their usual walking pace on a touch‐screen questionnaire at the baseline assessment visit. The question asked was “How would you describe your usual walking pace?” and they could select one of the three following options: brisk (>4 miles/h), average (3–4 miles/h), and slow walking pace (<3 miles/h), as described elsewhere.^24^, ^28^ Grip strength was measured using a Jamar J00105 hydraulic hand dynamometer. Isometric grip force was assessed from single 3‐s maximal grip efforts of the right and left sides with participants seated upright with their elbow by their side flexed at 90° so that their forearm was facing forward and resting on an armrest.^29^ In this study, absolute grip strength values were sex‐specific values. It, then, was categorized into tertiles, which were graded as strong, average, and weak. Walking pace and grip strength categories were combined as brisk‐strong, brisk‐average, brisk‐weak, average‐strong, average‐average, average‐weak, slow‐strong, slow‐average, and slow‐weak. Participants who were brisk walkers with strong grip strength were used as a reference group.

### 2.4 Covariates

The covariates included age, fruit and vegetable intake, red meat intake, processed meat intake, body mass index (BMI), as well as categorical variables sex, ethnicity, deprivation index, smoking status, alcohol intake, total sedentary time, sleep duration, and multimorbidity.

Age was calculated from birth and baseline assessment dates; sex was self‐reported; ethnicity was self‐reported and categorized as white, South Asian, Black, Chinese, and mixed. The deprivation index, an area‐based measure of socioeconomic status, was derived from the postal code of residence using the Townsend deprivation score.^30^ Fruit and vegetable, red meat, and processed meat intakes were recorded using a touch screen questionnaire asking about the reported frequency of consumption. Smoking status was categorized into never, former, and current. Alcohol intake was self‐reported and categorized as daily or almost daily, 3–4 times a week, once or twice a week, 1–3 times a month, special occasions only, and never. Total sedentary time was self‐reported and derived by combined TV viewing, leisure PC screen time, and time spent driving hours per day during leisure time. Participants were asked: “In a typical day, how many hours do you spend watching TV?”; “In a typical day, how many hours do you spend using the computer? (Do not include using a computer at work)”; and “In a typical day, how many hours do you spend driving?” Sedentary time was categorized as low (0–4 h), middle (5–6 h), and high (7–20 h). Sleep duration was self‐reported and classified as short sleep (<7 h/day), normal sleep (7–9 h/day), and long sleep (>9 h/day).^31^ BMI was calculated from weight (kg) divided by the square of height (m). The World Health Organization's criteria were used to classify BMI into categories of underweight (<18.5 kg/m^2^), normal weight (18.5–24.9 kg/m^2^), overweight (25–29.9 kg/m^2^), and obese (≥30 kg/m^2^). Multimorbidity was derived from participants who self‐reported chronic illnesses at baseline that included 43 medically diagnosed diseases. This covariate was categorized as a binary variable that as having no, or have 1, or more chronic illnesses. Additional details about these measurements can be found in the UK Biobank online protocol.^32^

### 2.5 Statistical analysis

Continuous variables are expressed as mean with SD and categorical variables are expressed as frequency and percentage. Cox‐proportional hazard models were used to investigate the associations of walking pace, grip strength, and the combination of walking pace and grip strength with incident T2D with follow‐up as the timeline variable. The results were reported as hazard ratios (HR) together with 95% confidence intervals (CI). The analyses were conducted within a 2‐year landmark period, which excludes any incident T2D occurring in the first 2 years of the follow‐up period and excluded all participants with prevalent diabetes (type 1 or type 2) or undiagnosed diabetes (HbA1c ≥ 48 mmol/mol), as well as those with missing data on walking pace, grip strength, and covariates.

The associations between walking pace, grip strength, and the combination of walking pace and grip strength and T2D incidence were adjusted for confounders with three models that included an increasing number of covariates. Model 1 (minimally adjusted model) was adjusted for age, sex, deprivation index, and ethnicity. Model 2 (lifestyle model) was adjusted for all variables in model 1 plus, fruit and vegetable intake, red meat intake, processed meat intake, smoking status, alcohol intake, total sedentary time, sleep time, and multimorbidity. Model 3 (adiposity model) was adjusted for all variables in model 2 plus BMI.

The additive interaction was conducted to test the interaction between walking pace and grip strength with incident T2D. The analyses were adjusted for confounders with three models as mentioned above. Relative excess risk due to interaction (RERI) was estimated to measure the additivity of the associations.^33^ The Kaplan–Meir survival estimate was also calculated.

The proportional hazard assumption was tested by Schoenfeld residuals. Statistical analyses were performed using the statistical software STATA 17 (StataCorp LP) and R v4.0.2. *p*‐values <0.05 were regarded as statistically significant.

### 2.6 Ethics statement

The UK Biobank study was approved by the North West Multi‐Centre Research Ethics Committee (Ref 11/NW/0382 on June 17, 2011) and all participants provided written informed consent to participate in the UK Biobank study. The study protocol is available online (http://www.ukbiobank.ac.uk/). This research has been conducted using the UK Biobank resource under application number 7155.

## 3 RESULTS

Of the 502 458 participants, 205 738 participants who had full data available for incident T2D, walking pace, grip strength, and covariates were included in this study (Figure S1). The median follow‐up period was 5.4 years (interquartile range: 4.8–6.5) after excluding the first 2 years. Over the follow‐up period, 5082 (2.5%) participants were diagnosed with T2D (2159 women and 2923 men). The person‐year incidence of T2D was 4.4 cases per 1000 person‐years, as shown in Table S1. Schoenfeld residuals test suggested that the proportional hazard assumption was not violented (*p*‐values = 0.17–0.27).

Table 1 presents the general characteristics of the participants by the combined walking pace and grip strength. Compared to other categories, individuals who were brisk‐strong were younger and were more likely to have lower deprivation. They were more likely to be non‐smokers, have more frequent alcohol consumption, lower red and processed meats intake, and lower sedentary time spent and shorter sleep duration. They were also more likely to have a strong hand‐grip strength than participants in other categories. Most individuals who were brisk‐strong had a BMI between normal weight and overweight categories. Cohort characteristics by sex are shown in Tables S2 and S3.

**TABLE 1:** Cohort characteristics of participants by the combined walking pace and grip strength

Characteristics	Overall	Brisk			Average			Slow		
		Strong	Average	Weak	Strong	Average	Weak	Strong	Average	Weak
Women, *n* (%)	115 139 (56.0)	18 135 (55.1)	14 936 (54.5)	13 027 (55.8)	17 399 (54.5)	19 215 (55.6)	23 963 (58.2)	1286 (55.0)	1932 (56.5)	5246 (61.0)
Men, *n* (%)	90 599 (44.0)	14 803 (44.9)	12 474 (45.5)	10 323 (44.2)	14 537 (45.5)	15 338 (44.4)	17 223 (41.8)	1054 (45.0)	1486 (43.5)	3361 (39.1)
Age, years (mean, SD)	56.4 ± 8.1	52.6 ± 7.7	55.9 ± 7.8	58.0 ± 7.6	53.8 ± 8.0	57.1 ± 7.8	59.2 ± 7.4	55.4 ± 7.9	58.4 ± 7.5	59.5 ± 7.2
Deprivation index tertiles, *n* (%)										
Lower deprivation	70 283 (34.2)	12 719 (38.6)	10 043 (36.6)	7960 (34.1)	11 399 (35.7)	12 086 (35.0)	12 780 (31.0)	625 (26.7)	882 (25.8)	1789 (20.8)
Middle deprivation	70 501 (34.3)	11 437 (34.7)	9623 (35.1)	8081 (34.6)	11 127 (34.8)	11 937 (34.6)	14 050 (34.1)	724 (30.9)	1035 (30.3)	2487 (28.9)
Higher deprivation	64 954 (31.6)	8782 (26.7)	7744 (28.3)	7309 (31.3)	9410 (29.5)	10 530 (30.5)	14 356 (34.9)	991 (42.4)	1501 (43.9)	4331 (50.3)
Ethnicity, *n* (%)										
White	197 740 (96.1)	32 179 (97.7)	26 761 (97.6)	22 572 (96.7)	30 765 (96.3)	33 268 (96.3)	38 922 (94.5)	2187 (93.5)	3202 (93.7)	7884 (91.6)
South Asian	2309 (1.1)	332 (1.0)	245 (0.9)	239 (1.0)	357 (1.1)	350 (1.0)	499 (1.2)	42 (1.8)	65 (1.9)	180 (2.1)
Mixed	3266 (1.6)	143 (0.4)	212 (0.8)	347 (1.5)	252 (0.8)	507 (1.5)	1280 (3.1)	41 (1.8)	81 (2.4)	403 (4.7)
Black	1930 (0.9)	256 (0.8)	146 (0.5)	146 (0.6)	488 (1.5)	334 (1.0)	339 (0.8)	60 (2.6)	57 (1.7)	104 (1.2)
Chinese	493 (0.2)	28 (0.1)	46 (0.2)	46 (0.2)	74 (0.2)	94 (0.3)	146 (0.4)	10 (0.4)	13 (0.4)	36 (0.4)
Lifestyles										
Smoking status, *n* (%)										
Never	114 642 (55.7)	19 609 (59.5)	15 963 (58.2)	13 541 (58.0)	17 467 (54.7)	18 782 (54.4)	22 791 (55.3)	1054 (45.0)	1521 (44.5)	3914 (45.5)
Previous	69 985 (34)	10 345 (31.4)	9169 (33.5)	7919 (33.9)	10 869 (34.0)	12 110 (35.1)	14 203 (34.5)	873 (37.3)	1312 (38.4)	3185 (37.0)
Current	21 111 (10.3)	2984 (9.1)	2278 (8.3)	1890 (8.1)	3600 (11.3)	3661 (10.6)	4192 (10.2)	413 (17.7)	585 (17.1)	1508 (17.5)
Alcohol intake, *n* (%)										
Daily or almost daily	41 510 (20.2)	7137 (21.7)	6290 (23.0)	5188 (22.2)	6332 (19.8)	6828 (19.8)	7579 (18.4)	389 (16.6)	549 (16.1)	1218 (14.2)
3–4 times a week	49 019 (23.8)	9351 (28.4)	7284 (26.6)	5863 (25.1)	7639 (23.9)	7944 (23.0)	8774 (21.3)	408 (17.4)	583 (17.1)	1173 (13.6)
Once or twice a week	54 708 (26.6)	8883 (27.0)	7320 (26.7)	5930 (25.4)	8987 (28.1)	9433 (27.3)	10 850 (26.3)	570 (24.4)	820 (24.0)	1915 (22.3)
1–3 times a month	23 207 (11.3)	3573 (10.9)	2797 (10.2)	2454 (10.5)	3921 (12.3)	4073 (11.8)	4701 (11.4)	270 (11.5)	429 (12.6)	989 (11.5)
Special occasions only	22 236 (10.8)	2546 (7.7)	2248 (8.2)	2303 (9.9)	3242 (10.2)	3866 (11.2)	5385 (13.1)	389 (16.6)	568 (16.6)	1689 (19.6)
Never	15 058 (7.3)	1448 (4.4)	1471 (5.4)	1612 (6.9)	1815 (5.7)	2409 (7.0)	3897 (9.5)	314 (13.4)	469 (13.7)	1623 (18.9)
Sleep categories, *n* (%)										
Normal (7–9 h per day)	152 715 (74.2)	25 551 (77.6)	20 858 (76.1)	17 303 (74.1)	24 280 (76.0)	25 916 (75.0)	30 002 (72.9)	1518 (64.9)	2185 (63.9)	5102 (59.3)
Short sleep (<7 h per day)	49 673 (24.1)	7159 (21.7)	6314 (23.0)	5796 (24.8)	7270 (22.8)	8125 (23.5)	10 358 (25.2)	717 (30.6)	1047 (30.6)	2887 (33.5)
Long sleep (>9 h per day)	3350 (1.6)	228 (0.7)	238 (0.9)	251 (1.1)	386 (1.2)	512 (1.5)	826 (2.0)	105 (4.5)	186 (5.4)	618 (7.2)
Total sedentary time, *n* (%)										
Low (<5 h/day)	94 159 (45.8)	17 398 (52.8)	13 965 (51.0)	11 956 (51.2)	13 507 (42.3)	14 588 (42.2)	17 666 (42.9)	763 (32.6)	1195 (35.0)	3121 (36.3)
Middle (5–6 h/day)	70 601 (34.3)	10 270 (31.2)	8970 (32.7)	7641 (32.7)	11 420 (35.8)	12 633 (36.6)	14 836 (36.0)	799 (34.2)	1167 (34.1)	2865 (33.3)
High (>6 h/day)	40 978 (19.9)	5270 (16.0)	4475 (16.3)	3753 (16.1)	7009 (22.0)	7332 (21.2)	8684 (21.1)	778 (33.3)	1056 (30.9)	2621 (30.5)
Diet and physical activity										
Fruit and vegetable intake, g/day (mean, SD)	327.1 ± 191.6	342.1 ± 192.4	348.6 ± 191.6	348.8 ± 195.3	310.8 ± 184.2	313.7 ± 181.3	320.4 ± 193.3	299.8 ± 196.7	304.0 ± 212.0	304.8 ± 210.1
Red meat intake, portion/week (mean, SD)	2.1 ± 1.4	2.0 ± 1.4	2.0 ± 1.4	2.0 ± 1.4	2.2 ± 1.4	2.2 ± 1.4	2.1 ± 1.5	2.3 ± 1.6	2.2 ± 1.6	2.2 ± 1.6
Process meat intake, portion/week (mean, SD)	1.8 ± 1.1	1.8 ± 1.0	1.7 ± 1.1	1.7 ± 1.1	1.9 ± 1.0	1.9 ± 1.0	1.9 ± 1.1	2.1 ± 1.1	2.0 ± 1.1	2.0 ± 1.1
Grip strength, kg (mean, SD)	30.7 ± 11.0	39.0 ± 10.3	31.2 ± 8.2	23.8 ± 7.6	38.8 ± 10.2	31.0 ± 8.1	22.9 ± 7.8	38.3 ± 10.1	30.6 ± 8.1	20.0 ± 8.3
Adiposity										
Body mass index (BMI), kg/m^2^ (mean, SD)	27.2 ± 4.6	26.0 ± 3.7	25.7 ± 3.7	25.6 ± 3.7	28.2 ± 4.6	27.8 ± 4.5	27.7 ± 4.5	31.5 ± 6.6	30.8 ± 6.1	30.3 ± 6.2
BMI category, *n* (%)										
Underweight (<18.5 kg/m^2^)	1043 (0.5)	157 (0.5)	195 (0.7)	226 (1.0)	75 (0.2)	98 (0.3)	218 (0.5)	2 (0.1)	9 (0.3)	63 (0.7)
Normal weight (18.5–24.9 kg/m^2^)	68 668 (33.4)	13 881 (42.1)	12 449 (45.4)	10 880 (46.6)	7870 (24.6)	9530 (27.6)	11 640 (28.3)	311 (13.3)	516 (15.1)	1591 (18.5)
Overweight (25.0–29.9 kg/m^2^)	89 097 (43.3)	14 286 (43.4)	11 632 (42.4)	9495 (40.7)	14 455 (45.3)	15 800 (45.7)	18 520 (45.0)	813 (34.7)	1175 (34.4)	2921 (33.9)
Obese (≥30.0 kg/m^2^)	46 930 (22.8)	4614 (14.0)	3134 (11.4)	2749 (11.8)	9536 (29.9)	9125 (26.4)	10 808 (26.2)	1214 (51.9)	1718 (50.3)	4032 (46.9)

*Note:* Data are presented as mean and standard deviation (SD) for continuous variables and as frequency and % for categorical variables.

Table 2 shows the association between walking pace and incident T2D. Compared to a brisk pace, individuals who reported average and slow pace had 33% (HR; 1.33 [95% CI, 1.24–1.43]) and 37% (HR; 1.37 [95% CI, 1.24–1.51]) higher risks of T2D, respectively. The magnitude and direction of T2D risk were similar in women and men (Table 2). Table 3 shows the association between grip strength and T2D incidence. The risk of T2D was 10% (HR; 1.10 [95% CI, 1.02–1.19]) and 22% (HR; 1.22 [95% CI, 1.13–1.31]) higher for those who had average and weak grip strength compared to strong grip strength, respectively. The magnitude and direction of T2D risk were similar in women and men (Table 3). The associations of walking pace and grip strength with T2D incidence for models 1 and 2 are shown in Tables S4 and S5, respectively.

**TABLE 2:** Association between walking pace and incident type 2 diabetes

Walking pace category	Event	Average pace	Slow pace
		HR (95% CI)	HR (95% CI)
Overall	5082	1.33 (1.24; 1.43)*	1.37 (1.24; 1.51)*
Women	2159	1.34 (1.20; 1.51)*	1.39 (1.19; 1.62)*
Men	2923	1.32 (1.21; 1.45)*	1.38 (1.21; 1.58)*

*Note:* Data are presented as hazard ratio (HR) and 95% CI. Brisk walking pace was the reference group (HR = 1.00). The fully adjusted model was adjusted for age, sex, ethnicity, deprivation index, smoking, fruit and vegetable intake, red meat intake, processed meat intake, alcohol intake, total sedentary time and sleep time, multimorbidity, and body mass index. All analyses were conducted using 2‐year landmark analyses and excluding participants with prevalent diabetes or unknown diabetes at baseline.

*p‐value < 0.0001.

**TABLE 3:** Association between grip strength and incident type2 diabetes

Grip strength tertiles	Event	Average	Weak
		HR (95% CI)	HR (95% CI)
Overall	5082	1.10 (1.02; 1.19)*	1.22 (1.13; 1.31)**
Women	2159	1.15 (1.02; 1.29)*	1.17 (1.05; 1.31)*
Men	2923	1.07 (0.97; 1.18)	1.25 (1.14; 1.38)**

*Note:* Data are presented as hazard ratio (HR) and 95% CI. Grip strength was sex‐specific values. Strong grip strength was the reference group (HR = 1.00). The fully adjusted model was adjusted for age, sex, ethnicity, deprivation index, smoking, fruit and vegetable intake, red meat intake, processed meat intake, alcohol intake, total sedentary time and sleep time, multimorbidity, and body mass index. All analyses were conducted using 2‐year landmark analyses and excluding participants with prevalent diabetes or unknown diabetes.

*p‐value < 0.05.

**p‐value < 0.0001.

**FIGURE 1:**
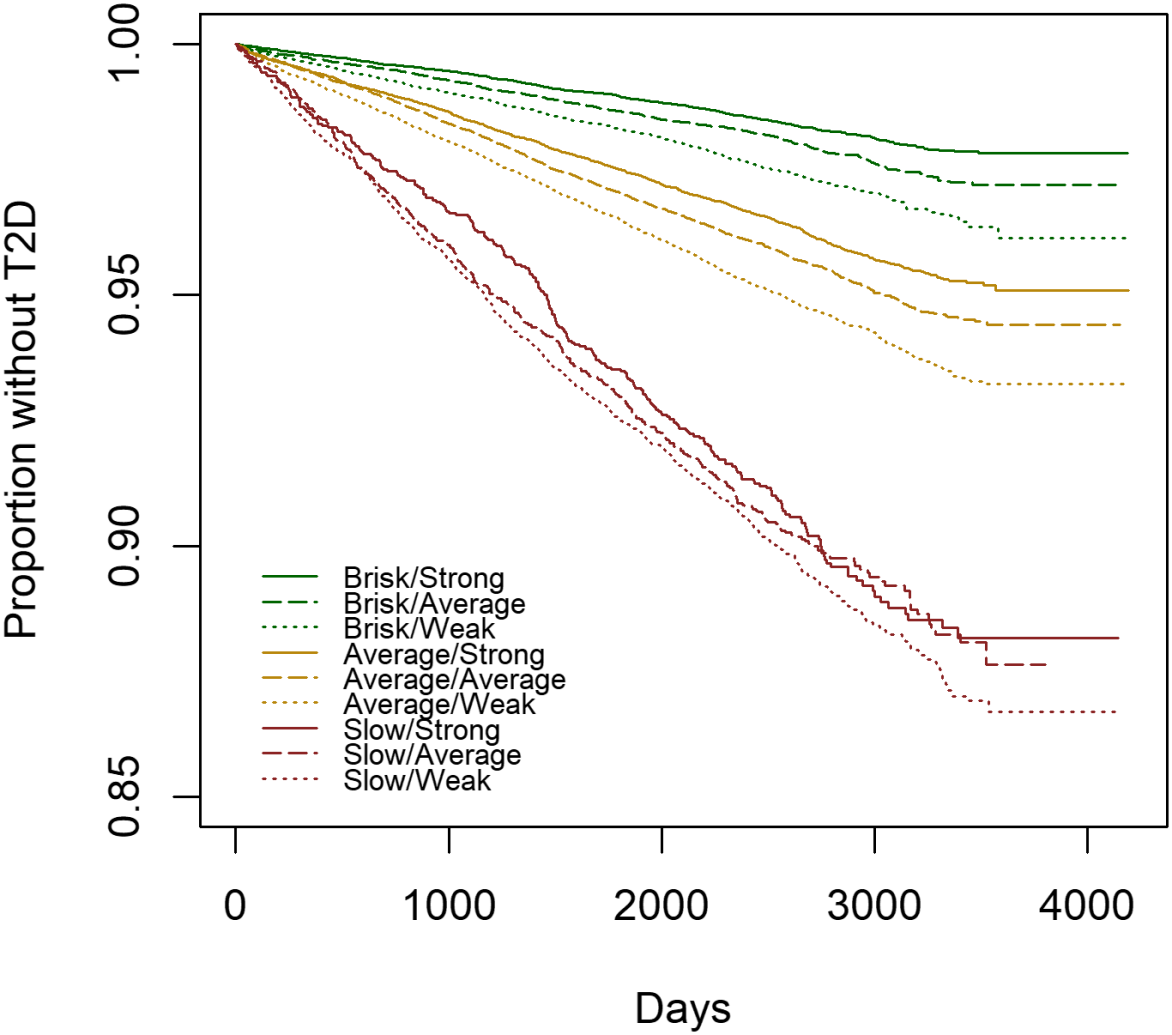
Kaplan–Meir survival estimates between combined walking pace and grip strength and incident type 2 diabetes. Data presented as the probability of survival during the follow‐up by the combined walking pace and grip strength. The analysis was conducted using 2‐year landmark analyses and excluding participants with prevalent diabetes or unknown diabetes at baseline

The combined associations between walking pace and grip strength and T2D incidence are shown in Table 4. After adjusting for all covariates (maximally adjusted model), the risk of T2D was higher for most categories compared to those classified as brisk‐strong. There was a modest, but negative, additive interaction (RERI; −0.06 [95% CI, −0.11, −0.01]). Brisk‐average and brisk‐weak had a 17% (HR; 1.17 [95% CI, 1.01–1.35]) and 34% (HR; 1.34 [95% CI, 1.16–1.55]) higher risk of T2D, respectively than those who were brisk‐strong. A similar pattern of the risk was also seen in those who were average‐strong (HR; 1.43 [95% CI, 1.27–1.62]), average‐average (HR; 1.51 [95% CI, 1.34–1.71]), and average‐weak (HR; 1.65 [95% CI, 1.47–1.86]). Individuals who were slow‐strong, slow‐average, and slow‐weak had a 49% (HR; 1.49 [95% CI, 1.22–1.82]), 58% (HR; 1.58 [95% CI, 1.32–1.89]), and 64% (HR; 1.64 [95% CI, 1.42–1.89]) higher risk of T2D, respectively. The associations were similar in women and men (Table 4). All models' associations of walking pace and grip strength with T2D incidence are shown in Table S6. The Kaplan–Meir survival estimates also showed that the slow walking pace and weak grip strength category had a lower probability of remaining free of T2D compared to the brisk‐strong category (Figure 1).

**TABLE 4:** Combined association of walking pace and grip strength with type 2 diabetes

	Walking pace	Overall			Women			Men		
		Brisk	Average	Slow	Brisk	Average	Slow	Brisk	Average	Slow
		HR (95% CI)	HR (95% CI)	HR (95% CI)	HR (95% CI)	HR (95% CI)	HR (95% CI)	HR (95% CI)	HR (95% CI)	HR (95% CI)
Grip strength	Strong	1.00 (Ref.)	1.43 (1.27; 1.62)	1.49 (1.22; 1.82)	1.00 (Ref.)	1.60 (1.30; 1.97)	1.38 (1.00; 1.89)	1.00 (Ref.)	1.34 (1.15; 1.57)	1.70 (1.32; 2.19)
	Average	1.17 (1.01; 1.35)	1.51 (1.34; 1.71)	1.58 (1.32; 1.89)	1.28 (1.00; 1.63)	1.66 (1.36; 2.04)	1.79 (1.36; 2.35)	1.12 (0.93; 1.34)	1.43 (1.23; 1.67)	1.47 (1.16; 1.87)
	Weak	1.34 (1.16; 1.55)	1.65 (1.47; 1.86)	1.64 (1.42; 1.89)	1.41 (1.11; 1.79)	1.65 (1.35; 2.02)	1.75 (1.39; 2.19)	1.31 (1.10; 1.57)	1.68 (1.44; 1.94)	1.59 (1.32; 1.92)

*Note:* Data are presented as HRs and their 95% CI. Brisk walkers with strong grip strength were used as the reference group (HR = 1.00). The model was adjusted for age, sex, ethnicity, deprivation index, smoking, fruit and vegetable intake, red meat intake, processed meat intake, alcohol intake, total sedentary time, sleep time, multimorbidity, and body mass index. All analyses were conducted using 2‐year landmark analyses and excluding participants with prevalent diabetes or unknown diabetes at baseline.

## 4 DISCUSSION

The main finding of this study is that the combination of slow walking pace and weak grip strength were associated with a higher risk of T2D, independently of sociodemographics, lifestyles, and adiposity, than either walking pace or grip strength alone. Additive interaction analysis showed only a slight overlap for the association between grip strength and walking pace, reassuring the importance of considering both markers. These findings have important public health relevance as both walking pace and grip strength are easy to measure and are inexpensive,^24^ and might be a useful tool to screen people at high risk of developing T2D.

Our findings agree with and extend the findings of previous studies on this topic. In the latest prospective cohort study investigating walking pace and T2D risk, conducted in Japan with a 3‐year follow‐up, data indicated that a fast walking pace was associated with a lower risk of T2D after adjusting for sociodemographic and lifestyle factors including BMI and systolic blood pressure.^34^ Therefore, our findings support this negative association but with a longer follow‐up time. On top of this, the current work confirmed the negative association between relative grip strength and incident T2D, seen previously,^35^ in the largest prospective cohort study so far.

A prospective cohort study, with 1085 older Japanese aged 65–89 years who were followed up for 10 years, investigated the combination of walking pace, grip strength, and standing balance to predict CVD, cancer, and all‐cause mortality. They indicated that adding grip strength to walking pace increased the ability to predict all‐cause mortality.^1^ Similarly, in our previous study, a large prospective cohort study in the UK Biobank with 406 834 participants with a follow‐up of 8.87 years, we found that CVD risk prediction was highest when walking pace was combined with grip strength.^24^ Although these previous studies have focused on CVD, it suggests that the combination of walking pace and grip strength are more strongly associated with health outcomes than in isolation. To the best of our knowledge, the combined association of walking pace and grip strength with the risk of developing T2D has not previously been investigated. Therefore, our findings indicated that the combination of walking pace and grip strength are associated more strongly (64% for slow‐weak) than either alone (37% for waking pace and 22% for grip strength), with the risk of developing T2D provides novel and important knowledge in this area.

To the best of our knowledge, our study was the first study that provided the additive interaction of the joint effect of two exposures between walking pace and grip strength on incident T2D. The results showed that the joint association was slightly lower (−6%) than the sum of their independent associations.

The strengths of the present study are that a large number of participants provided a sufficient sample size to undertake the analysis. We were also able to show that the associations were independent of various lifestyle factors and adiposity. Walking pace and grip strength are low cost, easy to administer, and, therefore, relatively simple to implement into clinical practice. Even though our study derived 45% of incident T2D linked to primary care data in the UK Biobank, these data have external validity for studying the prevalence and incidence of T2D.^36^ However, the present study has several limitations. Walking pace was self‐reported, so there might be bias in its measurement even though it also suggests the use of self‐report measures in the clinical setting. However, existing evidence shows that self‐reported walking speed is a good proxy of objectively measured walking or gait speed.^37^ The UK Biobank is not representative of the general population of the United Kingdom, including sociodemographic, physical, lifestyle, and health‐related characteristics of the general population. Although absolute risk would not be applicable to the general population, exposure‐disease risk estimates should be generalizable.^29^, ^38^ The observational nature of the study does not allow us to infer causality. Reverse causation may still be possible even though a 2‐year landmark analysis was conducted and individuals with diabetes at baseline and undiagnosed diabetes (HbA1c ≥ 48 mmol/mol) were excluded. However, we cannot exclude all residual confounding from our study.

To conclude, this is the first study to report the combined association of walking pace and grip strength and T2D incidence. The findings provide evidence that the combination of walking pace and grip strength are associated with T2D incidence, more than either alone, and this might be a practical approach to screening people who are at high risk of developing diabetes.

## 5 PERSPECTIVES

Physical activity is a crucial component of care in people with T2D,^39^ and markers of physical function are often associated with the risk of T2D. For example, low habitual walking pace and grip strength are associated with an increased risk of developing T2D. However, whether these 2 markers have an additive effect on the associations seen when employed in isolation. The current study found that both walking pace and grip strength are associated with incident T2D, but when added to the model in combination, the strength of the association is increased. This might be a practical approach to screening people who are at high risk of developing T2D.

## AUTHOR CONTRIBUTIONS

J.B., F.K.H., C.C‐M., and S.R.G. contributed to the study conception and design, advised on all statistical aspects, and interpreted the data. J.B., F.K.H., C.C‐M., and S.R.G. performed the statistical analyses. J.B., F.K.H., C.C‐M., and S.R.G. drafted the manuscript. J.B., S.P‐S., F.P‐R., F.K.H., C.C‐M., and S.R.G. reviewed the manuscript and approved the final version to be published. J.B., F.K.H., C.C‐M., and S.R.G. are the guarantors of this work and, as such, had full access to all the data in the study and take responsibility for the integrity of the data and the accuracy of the data analysis.

## CONFLICT OF INTEREST

The authors report no conflict of interest.

## Supporting information


Appendix S1

## Data Availability

The data that support the findings of this study are available from UK Biobank but restrictions apply to their availability. These data were used under license for the current study, and so are not publicly available. The data are, however, available from the authors upon reasonable request and with permission of UK Biobank. Data can be requested from the UK Biobank (https://www.ukbiobank.ac.uk/).
