# Supplementary material for: Combined association of walking pace and grip strength with incident type 2 diabetes
Source: Scand J Med Sci Sports. 2022 Jun 3;32(9):1356–65. doi: 10.1111/sms.14197 (PMC9544034; doi:10.1111/sms.14197)
Supplement: Supplementary file 1 — Appendix S1 [file SMS-32-1356-s001.docx]

**SUPPLEMENTARY MATERIALS**

**Supplementary Table S1.** The person-year incidence of type 2 diabetes and time to follow-up for walking pace, grip strength and combined walking pace and grip strength

| **Exposures** | **Person-time** | **Failures** | **Incident case**  per 1,000 person-years (95% CI) | **Time to follow-up**  Median (95% CI) |
| --- | --- | --- | --- | --- |
| **Walking pace** |  |  |  |  |
| Brisk | 471,196.4 | 1,130 | 2.4 (2.3; 2.5) | 5.4 (4.8; 6.3) |
| Average | 601,409.4 | 3,067 | 5.1 (4.9; 5.3) | 5.4 (4.8; 6.3) |
| Slow | 79,085.9 | 885 | 11.2 (10.5; 12.0) | 5.4 (4.7; 6.3) |
| **Grip strength** |  |  |  |  |
| Strong | 387,626.2 | 1,347 | 3.5 (3.3; 3.7) | 5.6 (4.9; 7.0) |
| Average | 365,443.0 | 1,521 | 4.2 (4.0; 4.4) | 5.4 (4.8; 6.3) |
| Weak | 398,622.4 | 2,214 | 5.6 (5.3; 5.8) | 5.3 (4.7; 6.0) |
| **Combined walking pace and grip strength** |  |  |  |  |
| Brisk/Strong | 190,549.1 | 372 | 2.0 (1.8; 2.2) | 5.6 (4.9; 7.0) |
| Brisk /Average | 153,058.5 | 373 | 2.4 (2.2; 2.7) | 5.4 (4.8; 6.2) |
| Brisk /Weak | 127,588.8 | 385 | 3.0 (2.7; 3.3) | 5.3 (4.7; 6.0) |
| Average/Strong | 183,802.3 | 829 | 4.5 (4.2; 4.8) | 5.6 (4.9; 7.0) |
| Average /Average | 193,312.8 | 944 | 4.9 (4.6; 5.2) | 5.4 (4.8; 6.3) |
| Average /Weak | 224,294.3 | 1,294 | 5.8 (5.5; 6.1) | 5.3 (4.7; 6.0) |
| Slow/Strong | 13,274.8 | 146 | 11.0 (9.4; 12.9) | 5.6 (4.8; 7.0) |
| Slow /Average | 19,071.8 | 204 | 10.7 (9.3; 12.3) | 5.4 (4.8; 6.6) |
| Slow /Weak | 46,739.3 | 535 | 11.4 (10.5; 12.5) | 5.3 (4.7; 6.1) |
| **Total** | 1,151,691.7 | 5,082 | 4.4 (4.3; 4.5) |  |

Data is presented as cases per 1,000 person-years for type 2 diabetes incident rate and median with 95% CI for time for follow-up. The analysis was adjusted for age, sex, ethnicity, deprivation index, smoking, fruit and vegetable intake, red meat intake, processed meat intake, alcohol intake, total sedentary time, sleep time, multimorbidity and body mass index. The analysis was conducted using 2-years landmark analyses and excluding participants with prevalent diabetes or unknown diabetes at baseline.

**Supplementary Table S2**. Cohort characteristics of participants by combined walking pace and grip strength in women

| Cohort characteristics | Overall | Brisk | | | Average | | | Slow | | |
| --- | --- | --- | --- | --- | --- | --- | --- | --- | --- | --- |
|  |  | Strong | Average | Weak | Strong | Average | Weak | Strong | Average | Weak |
| Total participants, n (%) | 115,139 (100.0) | 18,135 (15.8) | 14,936 (13.0) | 13,027 (11.3) | 17,399 (15.1) | 19,215 (16.7) | 23,963 (20.8) | 1,286 (1.1) | 1,932 (1.7) | 5,246 (4.6) |
| Age, years (mean, SD) | 56.2 ± 8.0 | 52.2 ± 7.6 | 55.8 ± 7.7 | 58.3 ± 7.3 | 53.4 ± 7.9 | 57.0 ± 7.7 | 59.2 ± 7.2 | 55.0 ± 7.9 | 57.6 ± 7.5 | 59.1 ± 7.2 |
| **Deprivation Index**, n (%) |  |  |  |  |  |  |  |  |  |  |
| Lower deprivation | 39,042 (33.9) | 6,793 (37.5) | 5,449 (36.5) | 4,468 (34.3) | 6,077 (34.9) | 6,660 (34.7) | 7,588 (31.7) | 338 (26.3) | 501 (25.9) | 1,168 (22.3) |
| Middle deprivation | 39,764 (34.5) | 6,320 (34.9) | 5,256 (35.2) | 4,561 (35.0) | 6,048 (34.8) | 6,682 (34.8) | 8,282 (34.6) | 421 (32.7) | 610 (31.6) | 1,584 (30.2) |
| Higher deprivation | 36,333 (31.6) | 5,022 (27.7) | 4,231 (28.3) | 3,998 (30.7) | 5,274 (30.3) | 5,873 (30.6) | 8,093 (33.8) | 527 (41.0) | 821 (42.5) | 2,494 (47.5) |
| **Ethnicity**, n (%) |  |  |  |  |  |  |  |  |  |  |
| White | 110,689 (96.1) | 17,671 (97.4) | 14,545 (97.4) | 12,596 (96.7) | 16,759 (96.3) | 18,515 (96.4) | 22,802 (95.2) | 1,206 (93.8) | 1,796 (93.0) | 4,799 (91.5) |
| South Asian | 1,418 (1.2) | 212 (1.2) | 157 (1.1) | 140 (1.1) | 209 (1.2) | 228 (1.2) | 284 (1.2) | 22 (1.7) | 41 (2.1) | 125 (2.4) |
| Mixed | 1,582 (1.4) | 68 (0.4) | 108 (0.7) | 171 (1.3) | 116 (0.7) | 222 (1.2) | 608 (2.5) | 13 (1.0) | 48 (2.5) | 228 (4.4) |
| Black | 1,135 (1.0) | 164 (0.9) | 99 (0.7) | 91 (0.7) | 267 (1.5) | 191 (1.0) | 178 (0.7) | 35 (2.7) | 38 (2.0) | 72 (1.4) |
| Chinese | 315 (0.3) | 20 (0.1) | 27 (0.2) | 29 (0.2) | 48 (0.3) | 59 (0.3) | 91 (0.4) | 10 (0.8) | 9 (0.5) | 22 (0.4) |
| **Lifestyles** |  |  |  |  |  |  |  |  |  |  |
| **Smoking** **status**, n (%) |  |  |  |  |  |  |  |  |  |  |
| Never | 68,999 (59.9) | 11,210 (61.8) | 9,162 (61.3) | 7,899 (60.6) | 10,286 (59.1) | 11,424 (59.5) | 14,548 (60.7) | 652 (50.7) | 1,020 (52.8) | 2,798 (53.3) |
| Previous | 35,933 (31.2) | 5,395 (29.8) | 4,719 (31.6) | 4,196 (32.2) | 5,390 (31.0) | 6,069 (31.6) | 7,415 (30.9) | 438 (34.1) | 616 (31.9) | 1,695 (32.3) |
| Current | 10,207 (8.9) | 1,530 (8.4) | 1,055 (7.1) | 932 (7.2) | 1,723 (9.9) | 1,722 (9.0) | 2,000 (8.4) | 196 (15.2) | 296 (15.3) | 753 (14.4) |
| **Alcohol** **intake**, n (%) |  |  |  |  |  |  |  |  |  |  |
| Daily or almost daily | 18,419 (16.0) | 3,299 (18.2) | 2,785 (18.7) | 2,372 (18.2) | 2,805 (16.1) | 2,938 (15.3) | 3,328 (13.9) | 156 (12.1) | 232 (12.0) | 504 (9.6) |
| 3-4 times a week | 24,260 (21.1) | 4,774 (26.3) | 3,624 (24.3) | 2,946 (22.6) | 3,668 (21.1) | 3,829 (19.9) | 4,384 (18.3) | 197 (15.3) | 261 (13.5) | 577 (11.0) |
| Once or twice a week | 30,822 (26.8) | 4,982 (27.5) | 4,134 (27.7) | 3,454 (26.5) | 4,830 (27.8) | 5,276 (27.5) | 6,330 (26.4) | 275 (21.4) | 449 (23.2) | 1092 (20.8) |
| 1-3 times a month | 15,323 (13.3) | 2,305 (12.7) | 1,787 (12.0) | 1,583 (12.2) | 2,561 (14.7) | 2,721 (14.2) | 3,216 (13.4) | 186 (14.5) | 289 (15.0) | 675 (12.9) |
| Special occasions only | 16,283 (14.1) | 1,820 (10.0) | 1,635 (11.0) | 1,656 (12.7) | 2,323 (13.4) | 2,855 (14.9) | 4,071 (17.0) | 271 (21.1) | 392 (20.3) | 1,260 (24.0) |
| Never | 10,032 (8.7) | 955 (5.3) | 971 (6.5) | 1,016 (7.8) | 1,212 (7.0) | 1,596 (8.3) | 2,634 (11.0) | 201 (15.6) | 309 (16.0) | 1,138 (21.7) |
| **Sleep** **categories**, n (%) |  |  |  |  |  |  |  |  |  |  |
| Normal (7-9 h per day) | 85,700 (74.4) | 14,278 (78.7) | 11,378 (76.2) | 9,587 (73.6) | 13,409 (77.1) | 14,419 (75) | 17,418 (72.7) | 858 (66.7) | 1,236 (64.0) | 3,117 (59.4) |
| Short sleep (<7 h per day) | 27,415 (23.8) | 3,705 (20.4) | 3,425 (22.9) | 3,288 (25.2) | 3,760 (21.6) | 4,477 (23.3) | 6,023 (25.1) | 375 (29.2) | 591 (30.6) | 1,771 (33.8) |
| Long sleep (>9 h per day) | 2,024 (1.8) | 152 (0.8) | 133 (0.9) | 152 (1.2) | 230 (1.3) | 319 (1.7) | 522 (2.2) | 53 (4.1) | 105 (5.4) | 358 (6.8) |
| **Total Sedentary time**, n (%) |  |  |  |  |  |  |  |  |  |  |
| Low (0-4 hrs/day) | 59,427 (51.6) | 11,105 (61.2) | 8,578 (57.4) | 7,340 (56.3) | 8,542 (49.1) | 9,191 (47.8) | 11,360 (47.4) | 482 (37.5) | 771 (39.9) | 2,058 (39.2) |
| Middle (5-6 hrs/day) | 38,457 (33.4) | 5,175 (28.5) | 4,667 (31.3) | 4,086 (31.4) | 6,059 (34.8) | 6,964 (36.2) | 8,576 (35.8) | 445 (34.6) | 676 (35.0) | 1,809 (34.5) |
| High (7-20 hrs/day) | 17,255 (15.0) | 1,855 (10.2) | 1,691 (11.3) | 1,601 (12.3) | 2,798 (16.1) | 3,060 (15.9) | 4,027 (16.8) | 359 (27.9) | 485 (25.1) | 1,379 (26.3) |
| **Diet and physical activity** |  |  |  |  |  |  |  |  |  |  |
| Fruit and vegetable intake, g/day (mean, SD) | 348.9 ± 188.3 | 364.8 ± 190.0 | 374.1 ± 187.3 | 377.1 ± 195.8 | 330.3 ± 184.0 | 333.9 ± 176.8 | 340.7 ± 184.9 | 316.7 ± 189.8 | 319.4 ± 213.2 | 324.3 ± 203.6 |
| Red meat intake, portion/week (mean, SD) | 2.0 ± 1.4 | 1.9 ± 1.3 | 1.8 ± 1.3 | 1.9 ± 1.3 | 2.0 ± 1.4 | 2.0 ± 1.4 | 2.0 ± 1.4 | 2.1 ± 1.5 | 2.1 ± 1.5 | 2.1 ± 1.5 |
| Process meat intake, portion/week (mean, SD) | 1.6 ± 1.0 | 1.5 ± 1.0 | 1.4 ± 1.0 | 1.5 ± 1.0 | 1.7 ± 1.0 | 1.7 ± 1.0 | 1.6 ± 1.0 | 1.8 ± 1.0 | 1.7 ± 1.0 | 1.7 ± 1.1 |
| Handgrip, kg (mean, SD) | 23.5 ± 6.2 | 30.6 ± 3.4 | 23.9 ± 1.4 | 17.8 ± 3.1 | 30.2 ± 3.1 | 23.9 ± 1.4 | 17.3 ± 3.5 | 29.9 ± 3.1 | 23.7 ± 1.4 | 15.2 ± 4.7 |
| **Adiposity** |  |  |  |  |  |  |  |  |  |  |
| Body mass index (BMI), kg/m^2^ (mean, SD) | 26.9 ± 5.0 | 25.2 ± 3.9 | 25.0 ± 3.7 | 25.1 ± 3.8 | 27.9 ± 5.1 | 27.6 ± 4.8 | 27.6 ± 4.8 | 32.3 ± 7.4 | 31.5 ± 6.7 | 30.9 ± 6.6 |
| **BMI category**, n (%) |  |  |  |  |  |  |  |  |  |  |
| Underweight (<18.5 kg/m^2^) | 837 (0.7) | 148 (0.8) | 162 (1.1) | 179 (1.4) | 67 (0.4) | 81 (0.4) | 154 (0.6) | 1 (0.1) | 9 (0.5) | 36 (0.7) |
| Normal weight (18.5-24.9 kg/m^2^) | 45,283 (39.3) | 9,722 (53.6) | 8,094 (54.2) | 6,923 (53.1) | ,5420 (31.2) | 6,166 (32.1) | 7,533 (31.4) | 188 (14.6) | 295 (15.3) | 942 (18.0) |
| Overweight (25.0-29.9 kg/m^2^) | 43,286 (37.6) | 6,233 (34.4) | 5,278 (35.3) | 4,535 (34.8) | 6,875 (39.5) | 7,889 (41.1) | 9,962 (41.6) | 366 (28.5) | 569 (29.5) | 1,579 (30.1) |
| Obese (≥30.0 kg/m^2^) | 25,733 (22.4) | 2,032 (11.2) | 1,402 (9.4) | 1,390 (10.7) | 5,037 (29.0) | 5,079 (26.4) | 6,314 (26.4) | 731 (56.8) | 1,059 (54.8) | 2,689 (51.3) |

Data is presented as mean and standard deviation (SD) for continuous variables and as frequency and % for categorical variables.

**Supplementary Table S3**. Cohort characteristics of participants by combined walking pace and grip strength in men

| Cohort characteristics | Overall | Brisk | | | Average | | | Slow | | |
| --- | --- | --- | --- | --- | --- | --- | --- | --- | --- | --- |
|  |  | Strong | Average | Weak | Strong | Average | Weak | Strong | Average | Weak |
| Total participants, n (%) | 90,599 (100.0) | 14,803 (16.3) | 12,474 (13.8) | 10,323 (11.4) | 14,537 (16.0) | 15,338 (16.9) | 17,223 (19.0) | 1,054 (1.2) | 1,486 (1.6) | 3,361 (3.7) |
| Age, years (mean, SD) | 56.5 ± 8.2 | 53.1 ± 7.9 | 56.0 ± 8.0 | 57.7 ± 8.0 | 54.3 ± 8.0 | 57.4 ± 7.9 | 59.3 ± 7.7 | 55.9 ± 8.0 | 59.4 ± 7.3 | 60.1 ± 7.2 |
| **Deprivation Index**, n (%) |  |  |  |  |  |  |  |  |  |  |
| Lower deprivation | 31,241 (34.5) | 5,926 (40.0) | 4,594 (36.8) | 3,492 (33.8) | 5,322 (36.6) | 5,426 (35.4) | 5,192 (30.2) | 287 (27.2) | 381 (25.6) | 621 (18.5) |
| Middle deprivation | 30,737 (33.9) | 5,117 (34.6) | 4,367 (35.0) | 3,520 (34.1) | 5,079 (34.9) | 5,255 (34.3) | 5,768 (33.5) | 303 (28.8) | 425 (28.6) | 903 (26.9) |
| Higher deprivation | 28,621 (31.6) | 3,760 (25.4) | 3,513 (28.2) | 3,311 (32.1) | 4,136 (28.5) | 4,657 (30.4) | 6,263 (36.4) | 464 (44.0) | 680 (45.8) | 1837 (54.7) |
| **Ethnicity**, n (%) |  |  |  |  |  |  |  |  |  |  |
| White | 87,051 (96.1) | 14,508 (98.0) | 12,216 (97.9) | 9,976 (96.6) | 14,006 (96.4) | 14,753 (96.2) | 16,120 (93.6) | 981 (93.1) | 1,406 (94.6) | 3,085 (91.8) |
| South Asian | 891 (1.0) | 120 (0.8) | 88 (0.7) | 99 (1.0) | 148 (1.0) | 122 (0.8) | 215 (1.3) | 20 (1.9) | 24 (1.6) | 55 (1.6) |
| Mixed | 1,684 (1.9) | 75 (0.5) | 104 (0.8) | 176 (1.7) | 136 (0.9) | 285 (1.9) | 672 (3.9) | 28 (2.7) | 33 (2.2) | 175 (5.2) |
| Black | 795 (0.9) | 92 (0.6) | 47 (0.4) | 55 (0.5) | 221 (1.5) | 143 (0.9) | 161 (0.9) | 25 (2.4) | 19 (1.3) | 32 (1.0) |
| Chinese | 178 (0.2) | 8 (0.1) | 19 (0.2) | 17 (0.2) | 26 (0.2) | 35 (0.2) | 55 (0.3) | 0 (0.0) | 4 (0.3) | 14 (0.4) |
| **Lifestyles** |  |  |  |  |  |  |  |  |  |  |
| **Smoking** **status**, n (%) |  |  |  |  |  |  |  |  |  |  |
| Never | 45,643 (50.4) | 8,399 (56.7) | 6,801 (54.5) | 5,642 (54.7) | 7,181 (49.4) | 7,358 (48.0) | 8,243 (47.9) | 402 (38.1) | 501 (33.7) | 1,116 (33.2) |
| Previous | 34,052 (37.6) | 4,950 (33.4) | 4,450 (35.7) | 3,723 (36.1) | 5,479 (37.7) | 6,041 (39.4) | 6,788 (39.4) | 435 (41.3) | 696 (46.8) | 1,490 (44.3) |
| Current | 10,904 (12.0) | 1,454 (9.8) | 1,223 (9.8) | 958 (9.3) | 1,877 (12.9) | 1,939 (12.6) | 2,192 (12.7) | 217 (20.6) | 289 (19.5) | 755 (22.5) |
| **Alcohol** **intake**, n (%) |  |  |  |  |  |  |  |  |  |  |
| Daily or almost daily | 23,091 (25.5) | 3,838 (25.9) | 3,505 (28.1) | 2,816 (27.3) | 3,527 (24.3) | 3,890 (25.4) | 4,251 (24.7) | 233 (22.1) | 317 (21.3) | 714 (21.2) |
| 3-4 times a week | 24,759 (27.3) | 4,577 (30.9) | 3,660 (29.3) | 2,917 (28.3) | 3,971 (27.3) | 4,115 (26.8) | 4,390 (25.5) | 211 (20.0) | 322 (21.7) | 596 (17.7) |
| Once or twice a week | 23,886 (26.4) | 3,901 (26.4) | ,3186 (25.5) | 2,476 (24.0) | 4,157 (28.6) | 4,157 (27.1) | 4,520 (26.2) | 295 (28.0) | 371 (25) | 823 (24.5) |
| 1-3 times a month | 7,884 (8.7) | 1,268 (8.6) | 1,010 (8.1) | 871 (8.4) | 1,360 (9.4) | 1,352 (8.8) | 1,485 (8.6) | 84 (8.0) | 140 (9.4) | 314 (9.3) |
| Special occasions only | 5,953 (6.6) | 726 (4.9) | 613 (4.9) | 647 (6.3) | 919 (6.3) | 1,011 (6.6) | 1,314 (7.6) | 118 (11.2) | 176 (11.8) | 429 (12.8) |
| Never | 5,026 (5.6) | 493 (3.3) | 500 (4.0) | 596 (5.8) | 603 (4.2) | 813 (5.3) | 1,263 (7.3) | 113 (10.7) | 160 (10.8) | 485 (14.4) |
| **Sleep** **categories**, n (%) |  |  |  |  |  |  |  |  |  |  |
| Normal (7-9 h per day) | 67,015 (74) | 11,273 (76.2) | 9,480 (76.0) | 7,716 (74.8) | 10,871 (74.8) | 11,497 (75) | 12,584 (73.1) | 660 (62.6) | 949 (63.9) | 1985 (59.1) |
| Short sleep (<7 h per day) | 22,258 (24.6) | 3,454 (23.3) | 2,889 (23.2) | 2,508 (24.3) | 3,510 (24.2) | 3,648 (23.8) | 4,335 (25.2) | 342 (32.5) | 456 (30.7) | 1116 (33.2) |
| Long sleep (>9 h per day) | 1,326 (1.5) | 76 (0.5) | 105 (0.8) | 99 (1.0) | 156 (1.1) | 193 (1.3) | 304 (1.8) | 52 (4.9) | 81 (5.5) | 260 (7.7) |
| **Total Sedentary time**, n (%) |  |  |  |  |  |  |  |  |  |  |
| Low (0-4 hrs/day) | 34,732 (38.3) | 6,293 (42.5) | 5,387 (43.2) | 4,616 (44.7) | 4,965 (34.2) | 5,397 (35.2) | 6,306 (36.6) | 281 (26.7) | 424 (28.5) | 1,063 (31.6) |
| Middle (5-6 hrs/day) | 32,144 (35.5) | 5,095 (34.4) | 4,303 (34.5) | 3,555 (34.4) | 5,361 (36.9) | 5,669 (37.0) | 6,260 (36.4) | 354 (33.6) | 491 (33.0) | 1,056 (31.4) |
| High (7-20 hrs/day) | 23,723 (26.2) | 3,415 (23.1) | 2,784 (22.3) | 2,152 (20.9) | 4,211 (29.0) | 4,272 (27.9) | 4,657 (27.0) | 419 (39.8) | 571 (38.4) | 1,242 (37.0) |
| **Diet and physical activity** |  |  |  |  |  |  |  |  |  |  |
| Fruit and vegetable intake, g/day (mean, SD) | 299.3 ± 192.3 | 314.1 ± 191.6 | 318.0 ± 192.3 | 313.1 ± 188.6 | 287.4 ± 181.8 | 288.3 ± 183.6 | 292.1 ± 201.2 | 279.2 ± 203.0 | 284.1 ± 208.9 | 274.5 ± 216.5 |
| Red meat intake, portion/week (mean, SD) | 2.3 ± 1.5 | 2.2 ± 1.4 | 2.2 ± 1.4 | 2.2 ± 1.5 | 2.3 ± 1.5 | 2.3 ± 1.4 | 2.3 ± 1.5 | 2.4 ± 1.7 | 2.4 ± 1.6 | 2.3 ± 1.6 |
| Process meat intake, portion/week (mean, SD) | 2.2 ± 1.0 | 2.1 ± 1.0 | 2.1 ± 1.1 | 2.1 ± 1.1 | 2.2 ± 1.0 | 2.2 ± 1.0 | 2.2 ± 1.0 | 2.4 ± 1.1 | 2.3 ± 1.0 | 2.3 ± 1.1 |
| Handgrip, kg (mean, SD) | 39.9 ± 8.8 | 49.4 ± 4.9 | 40.0 ± 1.9 | 31.3 ± 4.3 | 49.2 ± 4.7 | 39.9 ± 1.9 | 30.7 ± 4.7 | 48.6 ± 4.5 | 39.7 ± 1.9 | 27.5 ± 7 |
| **Adiposity** |  |  |  |  |  |  |  |  |  |  |
| Body mass index (BMI), kg/m^2^ (mean, SD) | 27.6 ± 4.0 | 27.0 ± 3.3 | 26.5 ± 3.4 | 26.2 ± 3.5 | 28.6 ± 4.0 | 28.0 ± 4.0 | 27.8 ± 4.1 | 30.5 ± 5.3 | 29.9 ± 5.0 | 29.3 ± 5.4 |
| **BMI category**, n (%) |  |  |  |  |  |  |  |  |  |  |
| Underweight (<18.5 kg/m^2^) | 206 (0.23) | 9 (0.1) | 33 (0.3) | 47 (0.5) | 8 (0.1) | 17 (0.1) | 64 (0.4) | 1 (0.1) | 0 (0.0) | 27 (0.8) |
| Normal weight (18.5-24.9 kg/m^2^) | 23,385 (25.8) | 4,159 (28.1) | 4,355 (34.9) | 3,957 (38.3) | 2,450 (16.9) | 3,364 (21.9) | 4,107 (23.9) | 123 (11.7) | 221 (14.9) | 649 (19.3) |
| Overweight (25.0-29.9 kg/m^2^) | 45,811 (50.6) | 8,053 (54.4) | 6,354 (50.9) | 4,960 (48.1) | 7,580 (52.1) | 7,911 (51.6) | 8,558 (49.7) | 447 (42.4) | 606 (40.8) | 1342 (39.9) |
| Obese (≥30.0 kg/m^2^) | 21,197 (23.4) | 2,582 (17.4) | 1,732 (13.9) | 1,359 (13.2) | 4,499 (31.0) | 4,046 (26.4) | 4,494 (26.1) | 483 (45.8) | 659 (44.4) | 1343 (40.0) |

Data is presented as mean and standard deviation (SD) for continuous variables and as frequency and % for categorical variables.

**Supplementary** **Table** S**4.** Association between walking pace and type 2 diabetes incidence

| Walking pace category | Event | Average pace | Slow pace |
| --- | --- | --- | --- |
|  |  | HR (95% CI) | HR (95% CI) |
| **Overall** | 5,082 |  |  |
| Model 1 |  | 1.97 (1.84; 2.11)^**^ | 3.80 (3.47; 4.16)^**^ |
| Model 2 |  | 1.72 (1.61; 1.85)^**^ | 2.57 (2.34; 2.83)^**^ |
| **Women** | 2,159 |  |  |
| Model 1 |  | 2.15 (1.92; 2.40)^**^ | 4.64 (4.05; 5.32)^**^ |
| Model 2 |  | 1.83 (1.63; 2.04)^**^ | 3.00 (2.60; 3.46)^**^ |
| **Men** | 2,923 |  |  |
| Model 1 |  | 1.87 (1.71; 2.04)^**^ | 3.27 (2.89; 3.7)^**^ |
| Model 2 |  | 1.67 (1.52; 1.82)^**^ | 2.28 (2.01; 2.59)^**^ |

^*^p-value <0.05

^**^p-value <0.0001

Data is presented as hazard ratio (HR) and 95% CI. Brisk walking pace was the reference group (HR=1.00). Model 1 (minimally adjusted model) was adjusted for age, sex, deprivation index and ethnicity. Model 2 (lifestyle model) was adjusted for all variables in model 1 plus, fruit and vegetable intake, red meat intake, processed meat intake, smoking status, alcohol intake, total sedentary time, sleep time and multimorbidity. All analyses were conducted using 2-years landmark analyses and excluding participants with prevalent diabetes or unknown diabetes at baseline.

**Supplementary Table S5.** Association between grip strength and type 2 diabetes incidence

| Grip strength tertiles | Event | Average | Weak |
| --- | --- | --- | --- |
|  |  | HR (95% CI) | HR (95% CI) |
| **Overall** | 5,082 |  |  |
| Model 1 |  | 1.07 (1.00; 1.16) | 1.30 (1.21; 1.40)^**^ |
| Model 2 |  | 1.04 (0.97; 1.12) | 1.18 (1.09; 1.26)^**^ |
| **Women** | 2,159 |  |  |
| Model 1 |  | 1.15 (1.02; 1.29)^*^ | 1.34 (1.20; 1.50)^**^ |
| Model 2 |  | 1.09 (0.97; 1.22) | 1.15 (1.03; 1.29)^*^ |
| **Men** | 2,923 |  |  |
| Model 1 |  | 1.03 (0.93; 1.13) | 1.28 (1.17; 1.41) |
| Model 2 |  | 1.01 (0.92; 1.11)^**^ | 1.20 (1.09; 1.31)^**^ |

^*^p-value <0.05

^**^p-value <0.0001

Data is presented as hazard ratio (HR) and 95% CI. Strong grip strength was the reference group (HR=1.00). Grip strength was sex-specific values. Model 1 (minimally adjusted model) was adjusted for age, sex, deprivation index and ethnicity. Model 2 (lifestyle model) was adjusted for all variables in model 1 plus, fruit and vegetable intake, red meat intake, processed meat intake, smoking status, alcohol intake, total sedentary time, sleep time and multimorbidity. All analyses were conducted using 2-years landmark analyses and excluding participants with prevalent diabetes or unknown diabetes at baseline.

**Supplementary** **Table** S**6**. Combined association of walking pace and grip strength with type 2 diabetes incidence

| Walking pace categories | Grip strength tertiles | Model 1 | | Model 2 | |
| --- | --- | --- | --- | --- | --- |
|  |  | HR (95% CI) | p-value | HR (95% CI) | p-value |
| **Overall** |  |  |  |  |  |
| Brisk | Strong | 1.00 (Ref.) |  | 1.00 (Ref.) |  |
|  | Average | 1.14 (0.99; 1.31) | 0.078 | 1.12 (0.97; 1.30) | 0.120 |
|  | Weak | 1.33 (1.15; 1.53) | <0.0001 | 1.26 (1.09; 1.45) | 0.002 |
| Average | Strong | 2.16 (1.91; 2.44) | <0.0001 | 1.9 (1.68; 2.15) | <0.0001 |
|  | Average | 2.17 (1.92; 2.45) | <0.0001 | 1.87 (1.66; 2.12) | <0.0001 |
|  | Weak | 2.40 (2.13; 2.70) | <0.0001 | 2.00 (1.77; 2.25) | <0.0001 |
| Slow | Strong | 4.64 (3.83; 5.62) | <0.0001 | 3.18 (2.62; 3.86) | <0.0001 |
|  | Average | 4.24 (3.57; 5.04) | <0.0001 | 2.88 (2.42; 3.43) | <0.0001 |
|  | Weak | 4.37 (3.81; 5.01) | <0.0001 | 2.83 (2.46; 3.26) | <0.0001 |
| **Women** |  |  |  |  |  |
| Brisk | Strong | 1.00 (Ref.) |  | 1.00 (Ref.) |  |
|  | Average | 1.27 (0.99; 1.61) | 0.055 | 1.23 (0.96; 1.56) | 0.095 |
|  | Weak | 1.48 (1.17; 1.88) | 0.001 | 1.36 (1.07; 1.73) | 0.011 |
| Average | Strong | 2.62 (2.13; 3.21) | <0.0001 | 2.25 (1.83; 2.76) | <0.0001 |
|  | Average | 2.66 (2.17; 3.26) | <0.0001 | 2.20 (1.79; 2.70) | <0.0001 |
|  | Weak | 2.69 (2.20; 3.28) | <0.0001 | 2.12 (1.73; 2.59) | <0.0001 |
| Slow | Strong | 5.70 (4.20; 7.73) | <0.0001 | 3.62 (2.66; 4.93) | <0.0001 |
|  | Average | 5.93 (4.56; 7.72) | <0.0001 | 3.88 (2.98; 5.07) | <0.0001 |
|  | Weak | 5.71 (4.60; 7.09) | <0.0001 | 3.46 (2.78; 4.32) | <0.0001 |
| **Men** |  |  |  |  |  |
| Brisk | Strong | 1.00 (Ref.) |  | 1.00 (Ref.) |  |
|  | Average | 1.07 (0.90; 1.28) | 0.450 | 1.07 (0.89; 1.28) | 0.482 |
|  | Weak | 1.25 (1.04; 1.50) | 0.017 | 1.21 (1.01; 1.45) | 0.036 |
| Average | Strong | 1.92 (1.65; 2.24) | <0.0001 | 1.72 (1.48; 2.01) | <0.0001 |
|  | Average | 1.92 (1.65; 2.23) | <0.0001 | 1.71 (1.46; 1.99) | <0.0001 |
|  | Weak | 2.27 (1.96; 2.63) | <0.0001 | 1.97 (1.70; 2.29) | <0.0001 |
| Slow | Strong | 4.11 (3.21; 5.28) | <0.0001 | 2.96 (2.30; 3.80) | <0.0001 |
|  | Average | 3.34 (2.64; 4.23) | <0.0001 | 2.33 (1.83; 2.95) | <0.0001 |
|  | Weak | 3.60 (3; 4.31) | <0.0001 | 2.44 (2.03; 2.94) | <0.0001 |

Data is presented as HRs and their 95% CI. Brisk walkers with strong grip strength were used as a reference group (HR = 1.00). Model 1 (minimally adjusted model) was adjusted for age, sex, deprivation index and ethnicity. Model 2 (lifestyle model) was adjusted for all variables in model 1 plus, fruit and vegetable intake, red meat intake, processed meat intake, smoking status, alcohol intake, total sedentary time, sleep time and multimorbidity. All analyses were conducted using 2-years landmark analyses and excluding participants with prevalent diabetes or unknown diabetes at baseline.


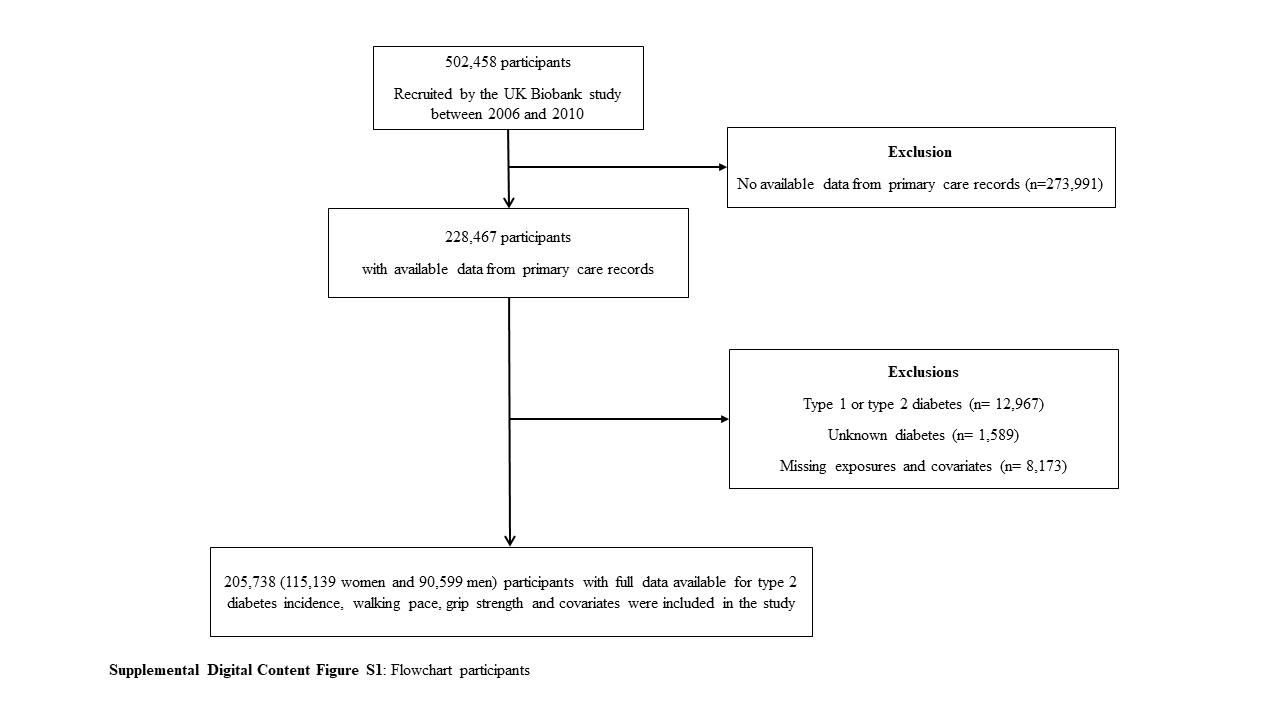


**Supplementary Figure S1**. Flowchart of participants throughout the study
